# Numerical and Experimental Study of a Lattice Structure for Orthopedic Applications

**DOI:** 10.3390/ma16020744

**Published:** 2023-01-12

**Authors:** Nikita Kharin, Pavel Bolshakov, Alex G. Kuchumov

**Affiliations:** 1 Institute of Mathematics and Mechanics, Kazan Federal University, 420008 Kazan, Russia; 2 Institute of Engineering, Kazan Federal University, 420008 Kazan, Russia; 3 Department Machines Science and Engineering Graphics, Tupolev Kazan National Research Technical University, 420111 Kazan, Russia; 4 Department of Computational Mathematics, Mechanics and Biomechanics, Perm National Research Polytechnic University, 614990 Perm, Russia; 5 Laboratory of Mechanics of Biocompatible Materials and Devices, Perm National Research Polytechnic University, 614990 Perm, Russia

**Keywords:** structural design, porous constructions, additive manufacturing, lattice structure, bone endoprosthesis

## Abstract

Prosthetic reconstructions provide anatomical reconstruction to replace bones and joints. However, these operations have a high number of short- and long-term complications. One of the main problems in surgery is that the implant remains in the body after the operation. The solution to this problem is to use biomaterial for the implant, but biomaterial does not have the required strength characteristics. The implant must also have a mesh-like structure so that the bone can grow into the implant. The additive manufacturing process is ideal for the production of such a structure. The study deals with the correlation between different prosthetic structures, namely, the relationship between geometry, mechanical properties and biological additivity. The main challenge is to design an endoprosthesis that will mimic the geometric structure of bone and also meet the conditions of strength, hardness and stiffness. In order to match the above factors, it is necessary to develop appropriate algorithms. The main objective of this study is to augment the algorithm to ensure minimum structural weight without changing the strength characteristics of the lattice endoprosthesis of long bones. The iterative augmentation process of the algorithm was implemented by removing low-loaded ribs. A low-loaded rib is a rib with a maximum stress that is less than the threshold stress. Values within the range (10, 13, 15, 16, 17, 18, 19 and 20 MPa) were taken as the threshold stress. The supplement to the algorithm was applied to the initial structure and the designed structure at threshold stresses σ_f_ = 10, 13, 15, 16, 17, 18, 19 and 20 MPa. A Pareto diagram for maximum stress and the number of ribs is plotted for all cases of the design: original, engineered and lightened structures. The most optimal was the designed “lightweight” structure under the condition σ_f_ = 17 MPa. The maximum stress was 147.48 MPa, and the number of ribs was 741. Specimens were manufactured using additive manufacturing and then tested for four-point bending.

## 1. Introduction

Nowadays, the number of operations involving arthroplasty is increasing day by day. However, some problems remain unresolved. One of the main particularities of arthroplasty is that the implant remains in the body after surgery. The solution to this problem is to make an implant using biomaterial, but biomaterial does not have the required strength characteristics [1,2]. The implant must also have a lattice structure in order for the bone material to grow through it. Additive manufacturing is an excellent solution for the production of such a structure.

Studies of the mesh structure produced by additive manufacturing techniques can improve the quality of endoprosthetics as a whole. Mesh structures printed in this way have sufficient strength and can also stimulate bone growth. Stimulation occurs, for example, by placing bone material inside [3,4,5]. Such structures would not be a complete substitute for a biomaterial prosthesis. However, there are currently a number of problems with the fabrication of biostructures.

Currently, the majority of endoprostheses are made of hard metal. Their main features are considered to be high biocompatibility and corrosion and wear resistance. However, the monolithic implants made of this material have higher stiffness than bone tissue. This property exposes the contact area between the endoprosthesis and the bone tissue to destruction [6]. The solution to this problem is the fabrication of mesh structures. Their design is based on factors such as strength and weight as well as a sufficient distance between the meshes for new tissue to take root. The retrofitting of mesh structures is still being investigated [7,8]. Studies have investigated the correlation between different prosthetic structures, namely, the relationship between geometry, mechanical properties and biological additivity. The main challenge is to design an endoprosthesis that will mimic the geometric structure of bone as well as meet the conditions of strength, hardness and stiffness.

Additive manufacturing allows the lattice structure to be manufactured in such a way that new tissues can grow and all mechanical parameters can be met, compared to the traditional manufacturing of lattice structures. The main disadvantage of this approach is considered to be the brittle properties of the structure and the deviation from the geometry during fusion [9,10,11,12,13]. There is also a trend toward manufacturing endoprostheses using composite materials [14]. Such systems combine calcium phosphate with ceramic or polymeric materials. This approach provides the necessary mechanical properties but is not suitable for individual implants. Selective laser melting technology is used to create an individual prosthesis. This method makes it possible to accurately produce an implant with a well-developed roughened surface [15,16,17].

Bone tissue is a complex anisotropic irregularly distributed regenerated material. Due to its heterogeneous structure, bone is constantly exposed to non-uniform loading, which results in a non-uniform stress–strain state. That is, such a structure is characterized by both overstressed areas and areas with insufficient loading. This fact should not be excluded when designing an endoprosthesis; an important task is to distribute the stress–strain state (SSS) evenly over the implant. In endoprosthesis design, a design with a regular distribution of cells is most commonly used. In this case, the structure can be considered as a certain topology. However, this approach does not result in all parts of the structure being evenly loaded. There is a question of modifying the structure in such a way as to minimize the amount of material used and to make uniform stress and strain distributions.

Previous studies have already addressed the design aspect of endoprostheses, achieving results to ensure that maximum stresses are reduced in an irregular, transversally isotropic design [14]. Nevertheless, there is no complex approach to the numerical and experimental study of lattice endoprosthesis design. Moreover, it is necessary to develop algorithms that enable us to construct the endoprosthesis with less weight and enhanced strength properties. Hence, the main objective of the present study is to provide an algorithm to obtain the minimal weight of the structure without decreasing the strength characteristics of the lattice endoprosthesis of long bones, as well as the verification of the numerical computations with experimental tests.

## 2. Materials and Methods

### 2.1. Brief Description of the Proposed Design Method

**Figure 1:**
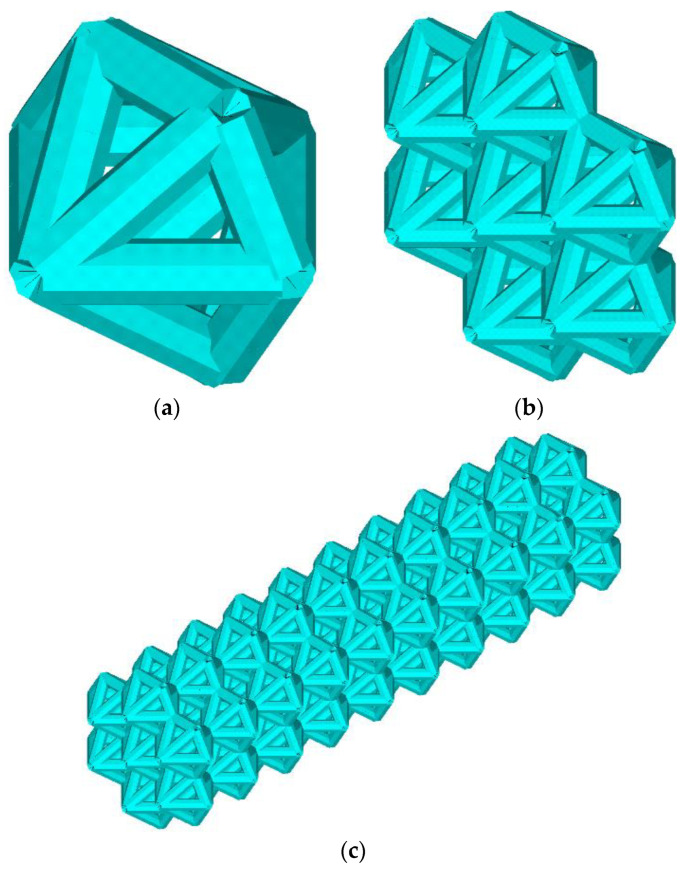
Geometry. Elementary cells (**a**), block (**b**), lattice endoprosthesis (**c**).

The lattice endoprosthesis (Figure 1c) is a set of blocks (Figure 1b). Each block consists of elementary cells (Figure 1a). In order to design a uniformly loaded product, we varied the dimensions of the blocks. The length of the blocks was varied using an influence function. Numerical experiments of the unit cell in compression and bending were performed to determine the influence function. The unit cell was described by the dimensionless parameter λ, which describes the ratio of its length to the radius of the circumscribed circle. The influence functions of parameter λ on the stress–strain state of the unit cell for compressive and bending loads have been obtained [14]. The idea of the algorithm was to determine the maximum normal stresses in each block and then modify the block lengths depending on the stresses while maintaining the product dimensions. The implant was optimized according to the previously proposed algorithm; the parameter vector was as follows: λ = (0.4, 0.4, 0.4, 0.4., 0.5, 0.8, 1.3, 1.7, 1.8, 2.3) [14]. The parameter vector λ is considered a vector with a 1xN dimension; each cell of the construction stores the value of the dimensionless parameter λ of the N-th block. Previously, we achieved a 53% reduction in maximum stresses compared to the original geometry [14]. However, we did not change the weight of the structure. The design Algorithm 1 presented in the previous paper is as follows:
**Algorithm 1** Endoprosthesis Design Algorithm**Input:** vector of parameters **λ** = [1, 1, 1, 1, 1, 1, 1, 1, 1, 1]**Output:** vector of parameters **λ** = [λ_1_, λ_2_,…, λ_9_, λ_10_]Create geometry according to parameter vector **λ**Create finite element meshApply workloads and boundary conditionsSSS problem solution**For** each **block**  Compute the maximum normal stresses in each block  Determine the vector of parameters **λ** depending on the influence functions of the elementary cell;**end**Check iteration stop condition

As mentioned earlier, the addition of the algorithm aims to reduce the weight of the product. The idea behind the addition of the algorithm is to remove the low-stress ribs of the prosthesis. A low-stress rib is a rib with a maximum stress that is lower than the threshold stress. The threshold stress is subjectively determined but does not exceed 4% of the yield strength of the material. However, first of all, the operating range of the algorithm must be defined as the endoprosthesis must be able to accommodate the placement of the bone material.

### 2.2. Mathematical Formulation of the Problem

**Figure 2:**
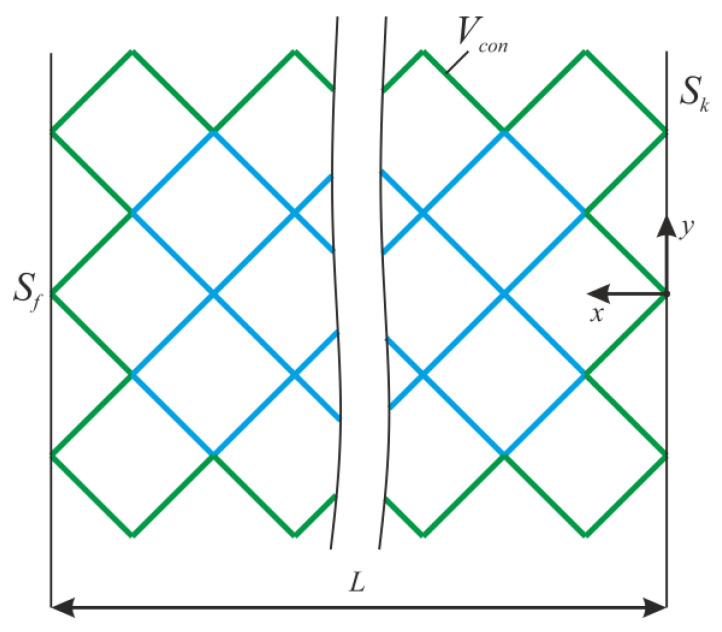
The design of lattice endoprosthesis.

The mechanical behavior of the endoprosthesis, within the framework of linear elasticity theory, can be described as follows:

(1) $$\nabla \cdot \tilde{\sigma }=0\;\;\;\;\;\;\;\;\;\;\;\;\;\;\;\;\forall \vec{x}\in V^{0}$$

(2) $$\tilde{\varepsilon }=\frac{1}{2}\left(\nabla \vec{u}+{\left(\nabla \vec{u}\right)}^{T}\right)\;\;\;\;\;\;\;\;\;\;\;\;\;\;\;\;\forall \vec{x}\in V^{0}$$

(3) $$\tilde{\sigma }=\tilde{\tilde{E}}:\tilde{\varepsilon }\;\;\;\;\;\;\;\;\;\;\;\;\;\;\;\;\forall \vec{x}\in V^{0}$$

(4) $$\vec{u}=0\;\;\;\;\;\;\;\;\;\;\;\;\;\;\;\;\forall \vec{x}\in S_{k}$$

(5) $$\tilde{\sigma }\cdot \vec{n}=\vec{F}\;\;\;\;\;\;\;\;\;\;\;\;\;\;\;\;\forall \vec{x}\in S_{f}$$

where *V*^0^—initial volume, *σ*—stress tensor, *ε*—strain tensor, *E*—stiffness tensor, *S_k_*—area with kinematic boundary conditions, *S_f_*—area with static boundary conditions, *u*—displacement vector, *n*—normal to the area with static boundary conditions, and *V_con_*—the constant region (Figure 2).

The design assumes that the endoprosthesis is manufactured using additive manufacturing. The material used is stainless steel PS4542A 17-4 PH: Young’s modulus 210 GPa, Poisson’s ratio 0.3, yield stress σ_Y_ = 500 MPa. The load in the numerical calculations was applied as follows: *F* = (−3, 1, 7) N, where −3 N is the compressive load in the x-direction [14].

### 2.3. Algorithm of Constructing A Lattice Structure

The algorithm is carried out in the entire construction area, except for the area *V_con_*. For *V_con_*, we consider the frame and support ribs of the endoprosthesis (Figure 2). After solving the stress–strain problem, the normal level of stress was determined at each i-th node of the rib.

Maximum normal stress at *i*-th rib σ_max_(i) corresponds to the maximum stress arising at the rib nodes.

The threshold stress σ_f_ was taken as the values on the interval [2% σ_Y_; 4% σ_Y_]. The rib where σ_max_(i) < σ_f_ was deleted, and after that, the stress–strain state problem was solved again. This process is iterative and stops when σ_max_(i) > σ_f_ at all ribs.

The addition to the Algorithm 2 of the lattice endoprosthesis design is as follows:
**Algorithm 2** Addition to Endoprosthesis Design Algorithm**Input:** vector of parameters **λ** = [λ_1_, λ_2_,…, λ_9_, λ_10_], edge numbers**Output:** edge numbersCreate geometry according to parameter vector **λ** and face numbersCreate finite element meshApply workloads and boundary conditionsDetermine the area of operation of the algorithm**while** flag  SSS problem solution  determine the maximum voltage σ_max_(i)  **if** σ_max_(i) < σ_f_ delete the i-th face  **else** go to **i + 1**  Check iteration stop condition**end**

### 2.4. FE Mesh Convergence

**Figure 3:**
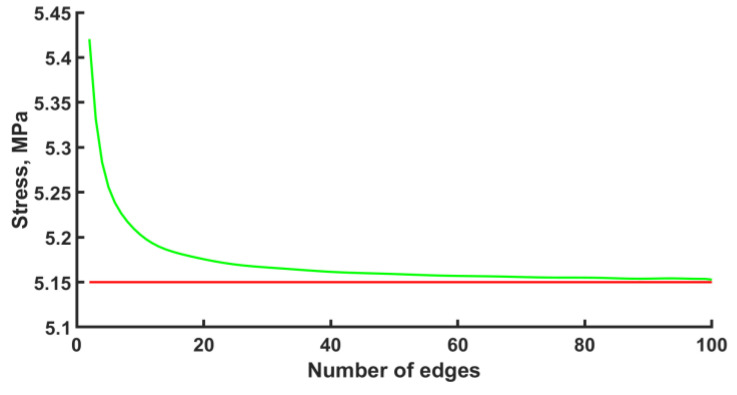
Mesh convergence plot (green line—approximation, red line—plateau).

The calculation was carried out with the ANSYS 2022 R1 software package. The finite element type was BEAM188 with quadratic approximation. The number of elements per edge was fifty. However, before the numerical calculation of the stress–strain state of an endoprosthesis, it is necessary to check the mesh convergence. The convergence of the mesh has been checked for one unit cell. The convergence of the mesh at the k-th node under the bending force F = −1 N can be seen in Figure 3. The k-th node is taken to be the node with the maximum stress that is not included in the area of boundary conditions. Based on the convergence of the mesh at the k-th node, in the subsequent design, for each edge, the number of elements equal to fifty was chosen. The error is 0.17%.

### 2.5. Experiment

Previously, in numerical calculations, it was shown that stress distribution does not depend on stiffness properties [18]. This can be explained by using a linear model in simulation. Such a fact allows the use of other materials in experiments. Of course, different materials have their own specific mechanical behavior, especially in the case of additive manufacturing. However, in an elastic region, this difference is minimal [19,20], especially if the same material specimens are used. That is why, because of economic expediency, the specimens were made from photopolymer resin.

**Figure 4:**
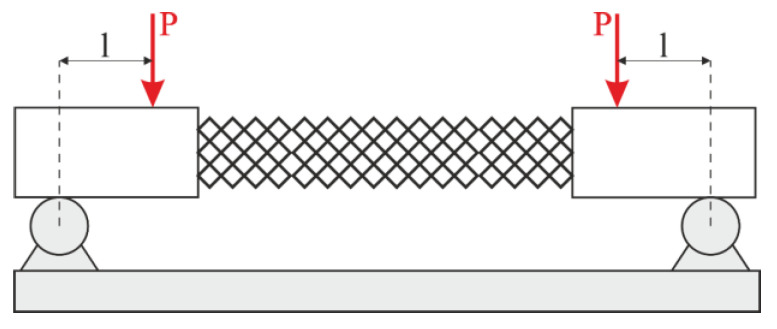
Loading scheme.

To provide pure bending, a four-point bending scheme was used. Points of force application were located on the arms (outside the structure). In this case, the constant bending moment acts on the specimen. The scheme of the four-point bending test is shown in Figure 4. There was no compression in the experiments. On one hand, adding compression complicates the experimental rig. On the other hand, the bending test is more informative for exploitation. In the case of compression loading, it is known that the failure mechanism of lattice structures begins with buckling. Then, the bending component appears and boosts the destruction. However, the pure bending test allows us to estimate the loading in ribs and nodes without the buckling effect.

To ensure metrological accuracy, the specimens were made on a scale of 2.5. The scale was determined based on the strength properties of the endoprosthesis in numerical experiments and the properties of the photopolymer resin. The scaling of the model is related to the low stiffness of the original endoprosthesis, as its length is 40 mm and the diameter of the rib is 0.4 mm. Additionally, the scaling effect was investigated.

Hence, the geometry of the endoprosthesis was reconstructed and manufactured using additive technologies. The samples were produced using an Anycubic Photon Mono X photopolymer 3d printer with a resolution of up to 10 µm. This was sufficient to produce the samples. Anycubic Basic photopolymer resin was used. The mechanical properties of the material were as follows: Young’s modulus 1.2 GPa, Poisson’s ratio 0.32, and tensile strength 23.4 MPa. Four-point bending tests were conducted according to the scheme shown in Figure 4. The specimen was mounted according to the bending plane with a numerical calculation scheme. Load curves were obtained, and the ultimate loads were analyzed.

## 3. Results

### 3.1. Lightening of the Original Construction

**Figure 5:**
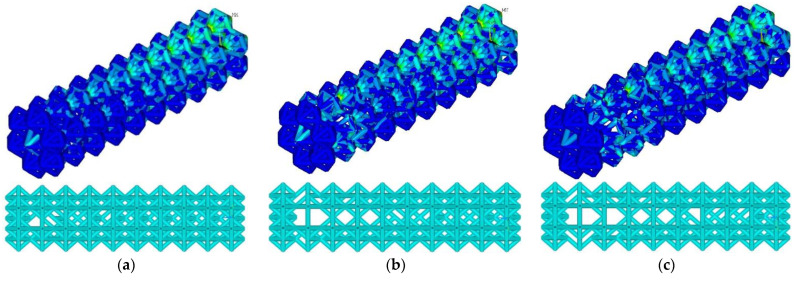
Von Mises stress distribution and endoprosthesis view with initial geometry at σ_f_ = 2% σ_Y_ (**a**), σ_f_ = 3% σ_Y_ (**b**), and σ_f_ = 4% σ_Y_ (**c**).

At the threshold stress σ_f_ = (2%, 3%, 4% σ_Y_), the maximum stresses in the structure were 155, 154, and 203 MPa, respectively (Figure 5). The number of ribs was 1015, 918, and 852, respectively. Compared to the initial geometry (1040 ribs), the number of ribs decreased to 2.4%, 11.7%, and 18%, respectively. As the threshold stress increases, a characteristic reduction in ribs in blocks 4–9 is observed. Maximal von Mises stress at σ_f_ = (2%, 3% σ_Y_) occurs in constrained zones (first block); von Mises stress at σ_f_ = 4% σ_Y_ occurs in the zone of the ninth block.

### 3.2. Lightening of the Updated Construction

**Figure 6:**
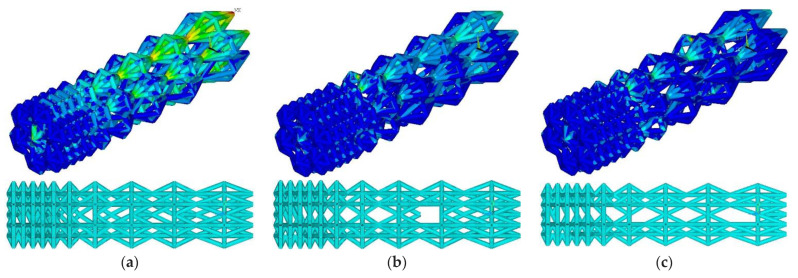
Von Mises stress distribution and endoprosthesis view with updated geometry at σ_f_ = 2% σ_Y_ (**a**), σ_f_ = 3% σ_Y_ (**b**), and σ_f_ = 4% σ_Y_ (**c**).

At the threshold stress σ_f_ = (2%, 3%, 4% σ_Y_), the maximum stresses in the designed construction were 71.5, 169.2, and 251.4 MPa, respectively (Figure 6). The number of ribs was equal to 911, 776, and 699, respectively. Compared to the initial geometry, the number of ribs decreased to 12.4%, 25.3%, and 32.7%, respectively.

### 3.3. Pareto Diagram

**Figure 7:**
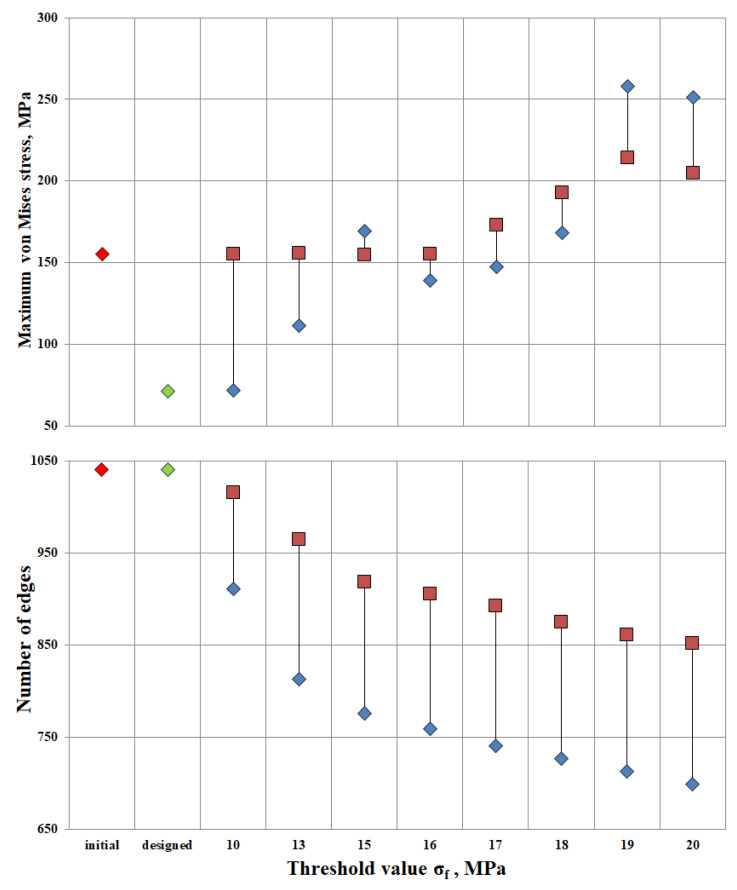
Pareto diagram; red rhombus—original structure, green rhombus—designed structure, red square—“lightweight” structure at λ = (1; 1; 1; 1; 1; 1; 1; 1; 1), and blue rhombus—“lightweight” structure at λ = (0.4, 0.4, 0.4, 0.4., 0.5, 0.8, 1.3, 1.7, 1.8, 2.3).

Additionally, the algorithm was applied to the original design and the developed design at threshold stresses σ_f_ = (10, 13, 15, 16, 17, 18, 19, 20 MPa). The Pareto diagram shows the results of the algorithm for different designs: stress and number of ribs in the original structure (the red rhombus in Figure 7), the constructed structure (the green rhombus in Figure 7), the “lightweight” original structure λ = (1; 1; 1; 1; 1; 1; 1; 1; 1) (the red square in Figure 7), and the “lightweight” constructed structure λ = (0.4, 0.4, 0.4, 0.4., 0.5, 0.8, 1.3, 1.7, 1.8, 2.3) (the blue rhombus in Figure 7).

The maximum stress in the original design is 155 MPa. Relaxation of the original design up to the threshold value σ_f_ = 16 MPa proceeds without a change in maximum stresses. Thus, having lightened the structure by 12.9%, the maximum stresses in the structure remained unchanged. Increasing the threshold to σ_f_ = 20 MPa, the number of ribs would decrease by 18%, but maximum tensions in the structure would increase by 32%. The value of σ_f_ = 16 MPa has been found to be the most appropriate threshold stress for relief of the original structure.

The maximum stress of the designed structure is 71.3 MPa. At design-induced lightening with the threshold value σ_f_ = 10 MPa, maximum stresses in the structure do not change. The number of ribs decreased by 12.3%. The lightweight design structure exhibits better strength properties than the original one. Up to the threshold value σ_f_ = 18 MPa, the maximum tension in the structure is 168.4 MPa at the reduction of the number of ribs by 30%.

The most optimal is the “lightened” design, provided σ_f_ = 17 MPa. The maximum stress was 147.48 MPa, and the number of ribs was 741. It can be concluded that by reducing the ribs by 28.7% relative to the original design, the stresses will decrease by 4.8%.

### 3.4. Experiment Results

**Figure 8:**
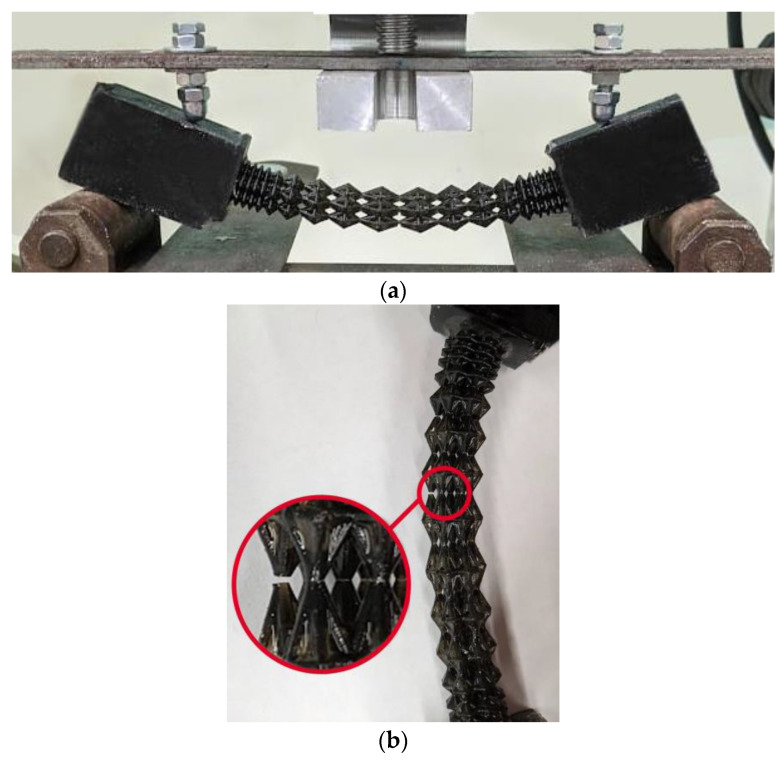

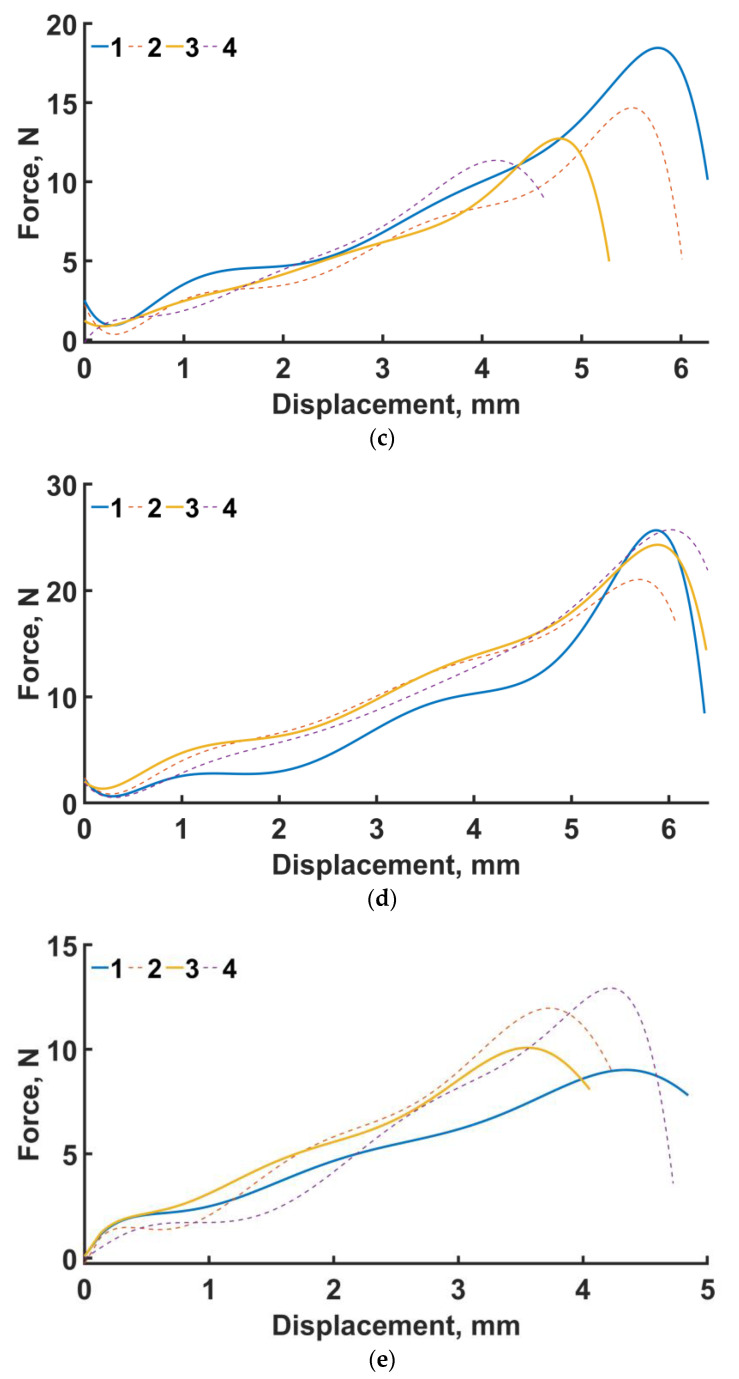
Four-point bending diagram (**a**), local fracture pattern (**b**), loading curve for the original structure (**c**), loading curve for the designed structure (**d**), and loading curve for the lightweight structure (**e**). Numbers 1–4 are samples 1–4.

Four samples of each design type were produced: initial, engineered, and lightweight at σ_f_ =17 MPa. Each specimen was tested according to the diagram shown in Figure 3. The length of the arm between the support and the applied load corresponds to l = 30 mm. The experimental setup with the designed specimen is shown in Figure 8a. The local fracture pattern of the designed sample is shown in Figure 8b.

The loading curves for all sample types are shown in Figure 8c–e. In the four-point bending experiments for the original geometry (see Figure 8c), the maximum force was 18.4 N and the displacement was 6 mm. For the engineered design (see Figure 8d), the maximum force was 25.7 N and the displacement was 5.8 mm. For the lightweight design (see Figure 8e), the displacement was 4.29 mm, with a maximum force of 12.9 N. The average maximum force for the original, engineered, and lighter structures at σ_f_ =17 MPa is 14.3, 24.1, and 11 N, respectively.

In percentage terms, the difference in average over all samples in terms of maximum force between the original and engineered geometry is 68.5%. In the numerical experiments, the maximum stresses of the designed structure are 53% lower than those of the original design.

The average maximum force of the lightened specimens is 23% less than that of the original design. In the numerical calculation, this percentage was 4.3% in favor of the lightened design.

## 4. Discussion

Numerous factors play an important role when designing novel implants [21]. Convenient methods showed a lot of limitations for effective implementation in clinical practice because of rapidly growing requirements from society, starting from performance properties and ending with ergonomics and aesthetic appearance. Additive manufacturing is a promising tool to overcome several limitations of traditional methods of production [22]. Despite the enormous costs, the constant stimulation of development is vital to meet the needs of an aging society [23]. Thanks to the adaptability of the method, the geometry can be tailored more precisely to the actual requirements of the patients, thus effectively counteracting the problem of wear protection, in particular [24].

On one hand, the FEM may be an appropriate instrument to examine the mechanical impacts of the changed prosthesis geometry. A number of past works dealing with it have been published [23,25,26].

On the other hand, topology optimization provides a novel strategy to optimize mechanical work by redesigning the dispersion of fabric in an assigned space. This dissemination depends on the specialized loading and boundary condition limitations that are subject to the objective aim to play down or maximize certain properties of the structure. It has been shown that topology optimization methods enable us to attain new implants with better biomechanics [27].

Hence, we can design and create a larger contact area between the prosthesis and the bone so that better adhesion can be realized. Increased bone-implant contact surface areas lead to a higher connection strength [28].

Experimental and numerical studies have shown good strength properties for the designed endoprosthesis. However, for the lightweight design, the average maximum load in the experiment was lower than that of the original specimens. This may be due to variations in cell geometry. Recently, similar conclusions were exhibited by Niutta et al. [29]. The removal of the ribs compromised the self-sustaining property of the whole structure. In other words, during local failure (Figure 8b) of the unit cell of both the original and engineered structures, the stress–strain state was redistributed to low-loaded areas of the structure, as previously indirectly shown by Smith et al. [30]. The lightweight structure does not have this property.

If we consider the lightweight and original designs, we note the fact that there is an overlap in the maximum forces of some samples. In the original design, the mean maximum stress of all samples was 14.3 ± 3.08, and in the case of the lightweight version, it was 10.99 ± 1.7. Looking at the median value of maximum stress in the original design, it was 13.7, and in the lightweight version, it was 10.99. Increasing the number of test specimens can give us the average maximum value for these types of specimens.

The incorrectness of the results also depends on the method used to print these samples [31]. In this study, the samples were printed using a photopolymer 3D printer. In the case of the lightweight design, there were difficulties in printing the internal (additional) supports. The stiffeners did not always perform their role correctly and, ultimately, the printed specimens had incorrect geometry [32]. The stiffener thickness causes the incorrectness of the geometry [33]. If the thickness is too thin, the stiffeners will bend under their own weight, causing the geometry of the design to be incorrect. This can be avoided by printing thicker stiffeners, but it is impossible to cut them out without disturbing the endoprosthesis structure.

Consideration of the heterogeneous properties ought to be the direction of future investigations. Research on the correlation between the implant degradation rate and bone growth may have great significance to bone fusion. Additionally, it is necessary to take into account the friction between bone and implant. In addition, the dynamic loading condition should be the main trend of future research [34].

## 5. Conclusions

An addition to the algorithm for the design of a long bone mesh implant has been proposed.

The iterative augmentation process of the algorithm was implemented by removing low-stress ribs. A low-stress rib is a rib with a maximum stress that is less than the threshold stress.

As to threshold stress σ_f_, the values in the range of (2% σ_Y_: 4% σ_Y_) (namely, 10, 13, 15, 16, 17, 18, 19, 20 MPa) were taken.

The yield stress of the 17-4PH material is equal to 600 ± 30 MPa. However, when making products using additive manufacturing techniques, there are difficulties in maintaining strength and mechanical properties. As such, yield stress σ_Y_ was taken to be equal to 500 MPa.

An endoprosthesis with parameter vector λ = (1; 1; 1; 1; 1; 1; 1; 1; 1) is considered the initial design. Updated construction of the endoprosthesis had parameter vector λ = (0.4, 0.4, 0.4, 0.4., 0.5, 0.8, 1.3, 1.7, 1.8, 2.3).

A Pareto diagram for maximum stress and the number of ribs was plotted for all design cases: original, designed and lightened structures.

The designed lightweight construction proved to be optimal under the condition σf = 17 MPa. The maximum stress was 147.48 MPa, and the number of ribs was 741.

The geometry of the endoprostheses was reconstructed, manufactured and tested for four-point bending. Four specimens of each type of construct were produced: initial, designed, and lightweight at σ_f_ =17 MPa. The original structures failed at a maximum load of 14.3 N, the designed structure at 24.1 N, and the lightened structure at 11 N.

## Data Availability

Data are available upon request.
